# Using GIS in a first national mapping of functional disability among older American Indians and Alaska natives from the 2000 census

**DOI:** 10.1186/1476-072X-5-37

**Published:** 2006-09-01

**Authors:** Margaret P Moss, Matthew C Schell, R Turner Goins

**Affiliations:** 1 University of Minnesota, School of Nursing, 308 Harvard Street, 6-138 Weaver-Densford Hall, Minneapolis, Minnesota, 55455, USA; 2 US Bureau of the Census, Geography Division, 4700 Silver Hill Rd, Washington, DC, 20233, USA; 3 West Virginia University, Department of Community Medicine and Center on Aging, PO Box 9127, Morgantown, West Virginia 26506-9127, USA

## Abstract

**Background:**

Geographical information systems (GIS) have been used mainly in understanding infectious diseases and environmental threats in health research. Here, GIS was used to examine patterns of functional disability as one *impact *of chronic disease in American Indians and Alaska Natives. The study purpose was to create the first national mapping of functional disability for AIANs using the 2000 U.S. Census.

**Results:**

American Indians and Alaska Natives over age 65 reported disability at a rate of 57.6% versus 41.9% for all people over 65 (*P *≤ 0.0001). Regional differences in levels and type of disability were evident.

**Conclusion:**

Maps help visualize those who might otherwise be 'lost' from the data. The significance of this study is that gerontologic programs and policies are data-driven, yet there is a lack of reliable national level data from US health systems on functional disability among American Indians and Alaska Natives. One study limitation was that Census questions regarding disability differed from traditional measures of activities of daily living and instrumental activities of daily living. An immediate policy recommendation would be to incorporate standard activities of daily living and instrumental activities of daily living language into future Census for a comprehensive, linked database for the future.

## Introduction

This study used Geographical Information Systems (GIS) in the examination of functional disability in American Indians and Alaska Natives. Geographical Information Systems (GIS) have not been used in nursing research "as a tool in the fight against disease"(p. 219) [1]. Mapping and spatial analysis have largely remained in the realm of public health.

The largest area for GIS and chronic disease mapping for instance, lies in cancer reporting [2,3]. Most of these appear to originate outside of the United States (US). In Hungary one study was conducted which used the National Public Health Service to establish monitoring in primary care facilities on selected chronic diseases [4]. Another study from the UK points to GIS as an important tool with which to 'join up' government and geographical data between agencies in tackling health issues [5]. These resources are lacking in the US.

The Centers for Disease Control (CDC) does track certain infectious diseases deemed as reportable and rely on health agencies to supply the data. However, there is no national health system or coordination between health facilities in the US, and therefore no ready database to track non-infectious, non-reportable chronic diseases. In fact, the GIS and health field internationally, is largely filled with infectious disease studies and those on the effects of environmental pollutants [6,7].

However, in the US where the public health focus has shifted from infectious diseases to chronic diseases [8], it will be important to translate this new technology for use in the chronic disease arena. American Indians as a subpopulation of the US often suffer from chronic diseases at rates often two to three times higher that for any other US group. Although much is known about the high levels of chronic disease rates among American Indian and Alaska Natives (AIANs) [8-11], little is known about functional disability among older AIANs. Chronic diseases affect AIANs at younger ages compared to the overall population and with some diseases, at higher rates. For example, young adults aged 25–44 years had an adjusted mortality rate of 26 per 100,000 for heart disease compared to 18 among same-aged Whites, and eight per 100,000 died of diabetes compared to three among Whites [11]. One tribe, the Pimas of Arizona, have the highest known prevalence of diabetes in the world [8]. Arthritis, one of the primary causes of disability is also high among older AIANs [12]. In addition, the high prevalence of risk factors for chronic disease, such as obesity, alcohol and tobacco use, has been well documented in AIAN populations [11].

Functional disability can be seen as an indication of the *impact *of chronic disease [13]. Measures of disability most widely used include limitations in activities of daily living (ADLs) and instrumental activities of daily living (IADLs) [14]. Functional status has been demonstrated as the single most important indicator for long-term care use [15]. One factor which has made it difficult to develop a national profile of functional disability both for the population at large and for AIAN is the heterogeneity of the population. As of December 2003, there were 562 federally recognized tribes, speaking over 200 languages [16], a number, which does not include state tribes, federally unrecognized tribes, as well as urban populations. American Indian communities are largely unlinked by any comprehensive data source around functional disability. In examining function and disability, this study employed the US Census 2000 as the link to locate and describe the prevalence of functional limitation in AIAN at the turn of the 21^st ^century.

To date, relatively few studies have examined functional disability among older AIANs [17-24]. Prevalence estimates indicate that older AIANs experience some of the highest disability rates compare to other U.S. racial groups. Data from the Medicare Current Beneficiary Survey found that 30.1% of AIANs had a limitation with at least one ADL compared to 17.0% of their White counterparts [17].

A study of 294 AIAN elders in Los Angeles, reported much lower percentages of functional disability, with toileting (4.9%) as the least frequent limitation and mobility (13.1%) as the highest [19]. In an attempt to gather national information on ADL limitations experienced by older AIANs, a survey was initiated by the National Resource Center on Native American Aging (NRCNAA). Moss and colleagues used the NRCNAA survey to conduct a secondary analysis focused on 90 older members of the Zuni tribe [18]. In this study, the mean number of ADL limitations was 1.4, with the most frequent ADL limitation in bathing (40%) and the least frequent in eating (11%). The results of this study indicated that the rates of disability were two to three times higher than those found in the Kramer study.

The problem addressed by this study is a lack of a national picture of the nature or extent of functional disability among older AIANs or whether there are differences by area of residence with regard to functional status. Mapping the occurrence and severity of functional disability among older AIANs can provide valuable information from which can guide further inquiry.

The CDC does have a state-by-state database on behavioral health, the Behavioral Risk Factor Surveillance System (BRFSS). The BRFFS is a telephone survey that gathers largely state level data related to chronic care [25]. The difficulty with this type of survey for AIAN elderly is in the method itself. More AIAN in Indian Country do *not *have a telephone than do [26]. When considering the elderly, the percentage drops. There are several tribes where elders either do not speak English or it is not their first language. For example, Navajo has almost 300,000 members where 75% still speak their language. Therefore, lack of phones, communication problems, and conceptual differences i.e. orality vs. literacy all point away from telephone use as an effective method for data gathering on AIAN elders, particularly in Indian Country. The census data is collected in person and in the mail with targeted efforts in Indian Country.

There has been one attempt to gather national information on ADLs on AIAN elderly specifically, a survey initiated by the National Resource Center on Native American Aging (NRCNAA). The primary author used their survey to conduct a secondary analysis focused on just one tribe [18]. In this study of 90 Zuni elders, the mean number of ADL limitations was 1.4 with the most frequent ADL limitation in bathing (40%) and the least frequent in eating (11%). Therefore, these elders should be considered disabled, with rates from two to three times higher than those found in the Kramer study. These widely divergent results point to the need to understand spatial, tribal and other differences in disability rates for AIANs.

There were severe limitations in the NRCNAA survey for use in research. First, it was conceived for administrative purposes. Other survey problems included: its use of convenience sampling, scaling errors in not providing a way to answer 'no' to having limitations or chronic conditions, and a long-term care question that only provided two options- nursing home or assisted living. Along with survey construction issues, there were methodological issues such as bias in forming the questions. For example, the survey asks about sweat lodge use and church attendance. Many tribes do neither; these are largely Plains Indian activities. Therefore, the challenge is to use more creative methods to get at a national inclusive picture of functional disability in AIANs, and their current long-term care options. When the author retooled the instrument and used random sampling the functional disability numbers dropped dramatically.

The purpose of this study was to create a first national map of functional disability for AIANs aged 65 years and older. It is imperative to gain a picture of the nature and extent of functional disability among older adults so that access points to care, long-term care options, and availability data will inform policy and health care decisions and funding will correspond with need. The specific aims of the current study were to use Census data to identify how many AIANs over age 65 report functional disability and map spatial patterns of disability to assess any regional and rural/urban differences in functional disability.

## Method

Geographical sampling and analyses were used in 'traditional' location-based methods [7] to provide visual analyses i.e. mapped evidence [27] for functional disability patterns in AIAN from the US Census 2000. The utility of basic mapping is presented in this paper. Simple overlays of Census data onto geography provide the basis for this study's methodology.

### Sample

The U.S. performs a Census of its population every ten years, counting total population and asking detailed questions about demographics, housing, and income. This study was a secondary data analysis of the 2000 U.S. Census. Persons included for this analysis were aged 65 years and over and self-identified as one race, "American Indian or Alaska Native."

The 2000 census was the first to allow the respondent to choose more than one race in identifying oneself. So as not to confound the results, the analysis included only those who chose one race. Further, as this was a 'first-look' we wanted to understand the picture of those who identify as American Indian 'more than any other group'. The reasoning being that someone may have had a great-grandmother who was Indian and so checks the box for American Indian but the risk factors, and geographical and cultural differences may not exist or may not be prominent. These disparities are what we are attempting to map for this article.

In 2000, 138,439 persons over age 65 identified themselves as AIAN and no other race. Institutional Review Board approval was given by the University of Minnesota.

### Measures

Data on disability status was derived from the Census "long form," which was sent to approximately one in six households in 2000. The Census distributes long forms randomly within geographic areas at rates which differ by population density. Rural areas are surveyed at rates higher than the nationwide average of one in six, and urban areas are sampled at rates lower than one in six [28]. This data is extrapolated by the Census Bureau to create data for the entire population, and the extrapolated data was used in the analysis. According to tables on the US Census Website, there were only 11,578 males and 22,466 females over 65 years who identified as one race, AIAN who lived alone. This is out of a total of almost 140,000 households with 65 yr olds and over who identified as one race and AIAN [29]. Over 75% of all of these elders live with others. We would reasonably expect that the one in six households with AIAN elders representing both genders answered these questions, or about 23,070.

In the Census questionnaire, ADL targeted queries are worded differently than are standard ADL and IADL items. Standard ADL items often ask whether the participant needs no assistance, some assistance, or complete assistance with the standard measures of walking, bathing, etc. The U.S Census uses "yes" and "no" as the choices for whether one experiences functional limitations. According to McBride, one or more ADL limitations are disabling and two or more are severely disabling [30]. One limitation of census disability data is that it reports counts of each disability within a unit of geography, so it is not possible to determine whether the same individual is appearing in the same counts. It is therefore not possible to address issues related to more than one disability. However, the census data does include one derived disability count, which is the total number of individuals responding affirmatively to any one or more of the disability questions. This derived count is utilized in Figures 2 and 5 of the analysis.

**Figure 4:**
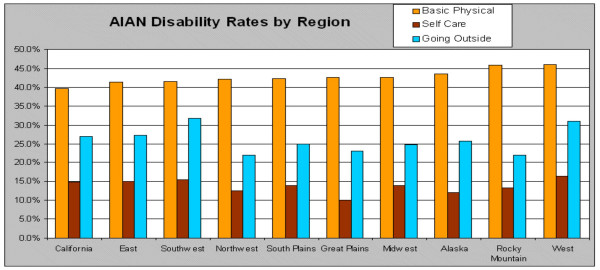
Distribution of older AIAN disability by region from the US Census 2000.

#### Impairment

The items in the U.S. Census used to define impairments included affirmative answers to question 16a ("Does this person have any of the following long-lasting conditions: Blindness, deafness, or a severe vision or hearing impairment") and 17a ("Because of a physical, mental, or emotional condition lasting 6 months or more, does this person have any difficulty in doing the following activities: Learning, remembering, or concentrating").

#### IADL loss

Answering "yes" to question 17 ("Because of a physical, mental, or emotional condition lasting 6 months or more, does this person have any difficulty in doing the following activities:") part c ("Going outside the home alone to shop or visit a doctor's office") and part d ("Working at a job or business") were used to define IADL loss.

#### ADL loss

Answering "yes" to question 16b ("Does this person have any of the following long-lasting conditions: A condition that substantially limits one or more basic physical activities such as walking, climbing stairs, reaching, lifting, or carrying") and 17b ("Because of a physical, mental, or emotional condition lasting 6 months or more, does this person have any difficulty in doing the following activities: Dressing, bathing, or getting around inside the home") was used to define ADL loss.

### Procedure

County-level data was extracted from Summary File 4 of the Census using a compact disk dataset issued by the Census Bureau. The data extracted was for tables PCT69 through PCT75 for AIANs over age 65, which is the dataset for AIAN answering "yes" to any question falling under number 16 or 17 on the long form pertaining to impairment, IADL loss, and ADL loss. The resulting tabular data was imported into the Geographic Information Systems (GIS) software program ArcView (Version 9, ERSI, 2005).

The geographical data in a GIS, such as a file containing counties of the U.S., commonly has attribute data associated with it. The table of disability data extracted from the Census contains one record per county, and the full dataset was brought into the GIS by joining each record to a county in the GIS. The resulting GIS dataset is richer than the original extracted data because, in addition to race, age, and disability information, the records have a spatial component that can be used in further analysis (i.e., cartographic visualization). Using the ADL/IADL data from the U.S. Census, spatial patterns of disability were mapped using varying levels of geographic overlays such as country, region, state and urban/rural and reservation. (For a more comprehensive description of GIS use, see [31]).

Several of the maps are reproduced in the results below. Because the study aim was to create a preliminary nationwide picture of elder AIAN disability levels, the maps attempt to present as much nationwide detail as possible, while at the same time not becoming so cluttered as to be unreadable. Counties were chosen as the unit of analysis for two reasons. First, in most cases it remains possible to distinguish individual counties at the scale reproduced in a typical journal article. Any greater level of detail, such as census tracts, would be unreadable. Second, counties were chosen to avoid constraints placed on census data to protect confidentiality. If the number of American Indians (or any race, respectively) is below 50 for a unit of analysis, no data is reported. The vast majority of the total AIAN elder population (89%) can be found in counties with over 50 AIAN. However, at any smaller unit of analysis, the number of elder AIAN included in the results falls precipitously.

**Figure 1:**
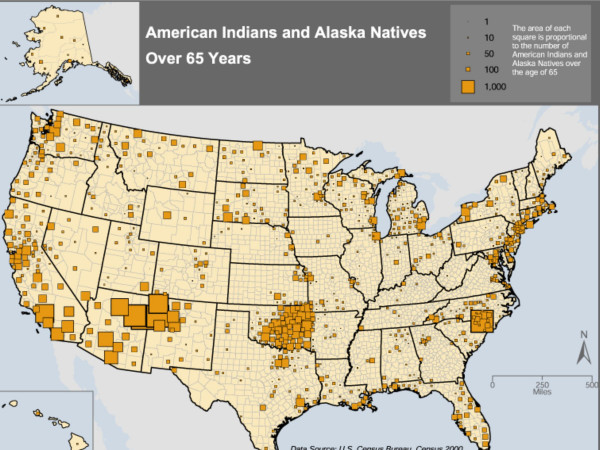
National distribution of AIANs 65 and over from the US Census 2000.

**Figure 2:**
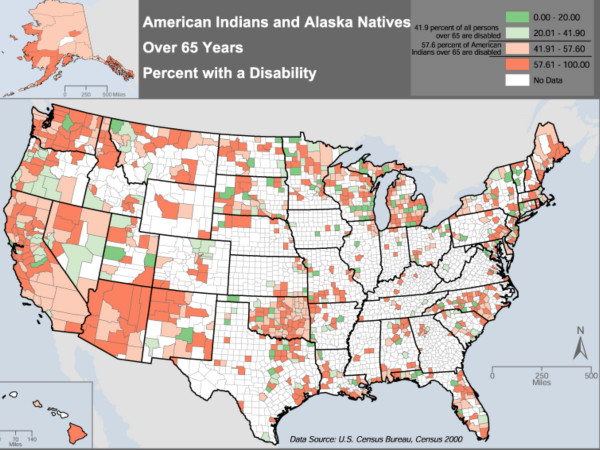
National distribution of AIANs 65 and over with a disability as compared to all persons over 65 with a disability from the US Census 2000.

**Figure 3:**
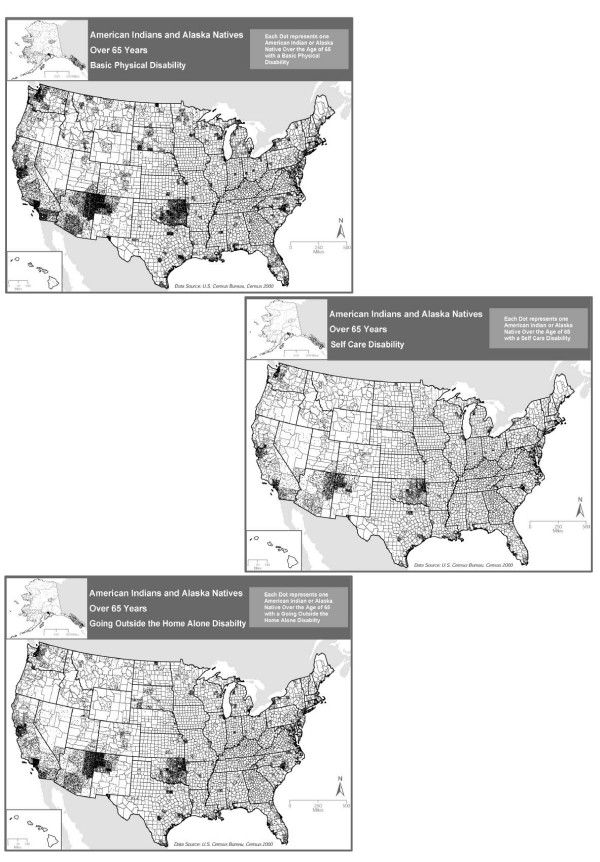
National distribution of disability in AIANs 65 and over for selected ADL/IADL from the US Census 2000: a) walking, b) bathing and c) getting out and about.

Three thematic map types are employed to visualize nationwide levels of impairment in AIAN elders. Figures 1 and 4 are known as proportional symbol maps, where the size of the symbol, in this case a square, is proportional to a quantity, in this case AIAN elders. Proportional symbol maps are most appropriate for visualizing counts of data, especially where the size of the data being mapped does not correspond to the spatial unit of analysis. In this case, small counties which are difficult to see may have large numbers of the variable being mapped. The second type of map, used in Figure 2, is a choropleth map in which the color of the county corresponds to the percent of the variable being mapped. Choropleth maps are most appropriate for normalized data, such as rates of disability. The third map type, employed in Figure 3, is a dot map. Dot maps create an impression of density and are most appropriate for showing distribution of a discrete population over space.

Raw counts of the number of AIANs age 65 and over, by county, are mapped in Figure 1 in order to give a general overview of the population distribution. The percent of AIAN elders who responded affirmatively to any one of the functional disability questions 16 or 17 in the census questionnaire are depicted in Figure 2. This is a derived quantity, and does not provide detail on differences between impairment, IADL loss, and ADL loss. In Figure 3 we attempt to provide more detail. Part "a" of Figure 3 maps the ADL loss corresponding to census question 16b ("Does this person have any of the following long-lasting conditions: A condition that substantially limits one or more basic physical activities such as walking, climbing stairs, reaching, lifting, or carrying"). Part "b" maps a second ADL loss, corresponding to census question 17b ("Because of a physical, mental, or emotional condition lasting 6 months or more, does this person have any difficulty in doing the following activities: Dressing, bathing, or getting around inside the home"). Lastly, part "c" maps the IADL loss corresponding to census question 17c ("Going outside the home alone to shop or visit a doctor's office").

Cartographic (mapping) visualization in turn suggests spatial patterns that can be explored further using other forms of quantitative analysis. Two clear patterns of functional disability that appear below are differences between regions of the country, and differences between rural and urban areas. In the numbers reported on regional differences, the disability rates are further aggregated into four regions, northeast, south, midwest, and west, as defined by the Census Bureau [32]. When reporting differences between urban and rural counties, urban counties were defined as those that are a part of a Metropolitan Statistical Area, and rural counties as those that are not. The U.S. Office of Management and Budget makes determinations on which areas qualify as Metropolitan Statistical Areas [33].

## Results

The number and location of AIANs over age 65 are mapped in Figure 1. According to the 2000 U. S. Census, there were 138,439 people who reported solely AIAN race and were aged 65 years and over. This number comes from the regular, non-sample, Census short form. However, this is an approximation; in the analysis of county-level data, there were 122,994 AIANs aged 65 and over because counties with less than 50 AIANs were not included. The sample used in the current study received the long form to include data on disability, resulting in a final sample size of *n *= 23,073. As illustrated in Figure 1, older AIANs reside largely in the central corridor of the U.S. and in western states. However, they can also be seen on the maps in large numbers in Michigan, for example, and along the eastern and southern coasts.

Figure 2 shows a comparison of disability for all persons over age 65 with otherwise-similar AIANs. For all persons in the U.S. over age 65, 41.9% have one or more disabilities, whereas 57.6% of same-aged AIANs have a disability (*P*≤0.0001). As shown on the map, the greater the proportion of AIANs in a particular county with a disability, the darker the shading on the map. It can be seen, for instance, that the island of Hawaii, the coastal and island regions of Alaska, northern Idaho, and California, Washington, New Mexico, Arizona and Nevada have large percentages of disabled AIANs.

Different levels and kinds of disability are found in different regions. One ADL limitation, the ability to walk, shows a noticeable concentration in the southwest (Figure 3a). Forty-three percent of AIAN elders in the west and south answered "yes" to this item, 42% in the Midwest, and 39% in the northeast (*P*≤0.0001). The ADL limitation of bathing, although noticeably less represented than the problem of walking, continues to be seen as a problem again in the southwest and variously across the country (Figure 3b). Although there are appears to be subregional differences, this ADL limitation does not vary significantly by region with 16% in the south and northeast, 15% in the west, and 12% in the Midwest (*P*≤0.0001). Finally, an example of an IADL limitation, getting out and about, is represented in Figure 3c. Twenty-nine percent of older AIANs report this limitation in the northeast, 28% in the west, 26% in the south, and 24% in the Midwest (*P*≤0.0001).

**Figure 5:**
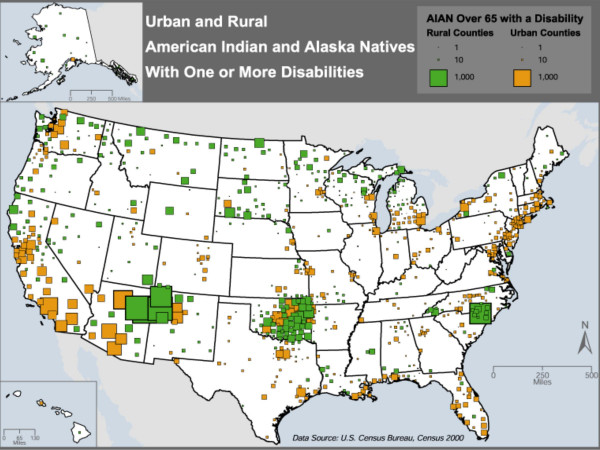
Geographic distribution of disability among AIANs 65 years and older showing urban vs. rural differences.

The final map, Figure 5, is a comparison of disability between urban and rural areas. Most reservations in the U.S. lie in rural areas. There are, however, a few in or near metropolitan centers. Figure 5 shows the number of AIANs over the age of 65 with at least one disability mapped by county. The size of each square is proportional to the number of AIANs with a disability, and the shade used for the squares distinguishes between urban and rural areas. Urban counties are defined as those that are part of a metropolitan statistical area, and rural counties are not part of a metropolitan statistical area. Nationwide, our analysis finds that 60.9% of AIAN elders in rural areas compared to 55.3% in urban areas report at least one disability (*P*≤0.0001). Urban areas of disability exist along the California and eastern coasts. Rural disability has the largest percentages in the Carolinas, Oklahoma, New Mexico, and Arizona. Texas, which has almost no rural representation for AIAN disability, shows disability in Houston, Dallas, San Antonio, and El Paso.

## Discussion

### Maps help visualize those who might otherwise be 'lost' from the data

While mapping the location and population density of all AIANs over age 65 provides important demographic information, it fails to direct attention to areas where disability rates are disproportionately high. The current study yielded several maps, which show complex spatial and occurrence information from AIANs across all 50 states including those that are home to the 560 tribes. These maps will be useful for understanding functional disability because they can be viewed instantly for trends and areas in need of services. For example, Texas is a state with vast rural areas and relatively few older AIANs. However, when functional disability is mapped by area of residence, there are large urban areas in Texas, which contain AIAN populations who have a high prevalence of functional disability. Furthermore, services, which focus on the special needs of AIAN elders, are limited in some large cities, such as Houston.

In another example, the lack of numbers of older AI/ANs in northern Idaho would not lead decision makers to allocate funding streams into the area for disability programs, yet these maps show that there is a disproportionate amount of AIAN disability for those who are there. Maps help visualize those who might otherwise be 'lost' from the data.

This study was important in linking geospatial/population data that might influence the placement of services that are specific to the density and type of disability in a certain geographic area. A New Zealand study found, that more attention must be paid to spatial information based in primary care to be more effective in planning services for disadvantaged populations [34]. A US study on marginalized populations found GIS an important tool in understanding the dynamics of population diversity and as a means of assessing marginal situations [35].

There are 4.1 million AIANs according to the 2000 Census [36]. Although they represent only 1.5 percent of the total U.S. population, this number is greater than the population of Los Angeles, California, by half a million. Previous research has shown that AIANs develop chronic diseases at earlier ages and die from them at earlier ages [11]. Thus, the tension between long-term care need and the inability of the health care system to deliver care where and when it is needed remains largely unresolved. The Indian Health Service, charged with providing care for AIANs, does not provide institutional long-term care such as nursing home care, assisted living or adult day care and does not report statistics on functional status. AIAN families largely carry the burden of providing care for disabled elders though they themselves have few resources.

There are some tribal grant opportunities offered by the IHS to develop solutions to the eldercare problem. However, these are relatively new and have only addressed a few tribes who are successful in the granting process. Without detailed national data on functional disability, such as the information provided in the current study, the extent of long-term care needs among older AIANs will remain largely unknown.

Limitations to the current study include issues related to the data available from the US Census. These include inability to fully access some AIAN communities, misidentification and related over- or under-representation, mobility of the group, and potential difficulty in answering the Census forms. Over-representation may occur when respondents incorrectly choose AIAN as their race on the forms; there is no verification of being AIAN. Anyone could in theory identify on the form as AIAN whether or not there was a genetic basis. Yet there are legal definitions which include being an enrolled member of a federally recognized tribe and not just having some degree of heritage (Title 25, US Code). The legal definition would apply to those who are eligible for care under IHS criteria. A related problem is in deciding whether to use one race or any that include American Indian. It was hypothesized that many elders would chose AIAN as their category i.e. one race. Therefore, in an attempt to make as clean a map as possible for a first look we settled on this category. Another problem is under-representation, which occurs when access, mobility and cultural problems preclude AIANs from successfully completing forms.

Further, the questions about disability are not standard ADL/IADL items. However, as a baseline from which to work, the Census data does allow a preliminary picture of some aspects of functional disability in this group.

More than 90 million Americans live with a chronic disease and of these, 25 million suffer from major limitations in activity caused by chronic diseases [8]. The looming burden of chronic disease and disability among AIANs [8,11] must become a priority in the U.S. health policy agenda. While the life span of AIANs has increased over the last century, although still several years behind U.S. Whites [11], AIANs are now living longer with functional disability.

The significance of this study is that gerontologic programs and policies are data-driven, yet there is a lack of reliable national level data from US health systems on functional disability among AIANs. This study provides a first look at function in a largely vulnerable, underserved and marginalized US population.

A seminal recommendation arising from this study is for the US Census to incorporate the accepted research and practice wording for ADLs and IADLs into the long form. In doing so, the US can begin to develop a comprehensive, linked database with which to monitor functional disability patterns temporally as well as spatially. This information could be translated and directly applicable in gerontology toward meeting the needs of any slice of the population. Additionally, the White House Conference on Aging also meets every decade (mid-decade) and would be able to incorporate such useable information in the formation of future gerontologic policy.

Future studies are needed to extend this information into modeling causative factors for functional disability linked to disease and environment, spatial relationships to relevant health care services and policy implications.
